# Risk of Cancer Mortality in Spanish Towns Lying in the Vicinity of Pollutant Industries

**DOI:** 10.5402/2012/614198

**Published:** 2012-09-27

**Authors:** Rebeca Ramis, Pablo Fernandez-Navarro, Javier Garcia-Perez, Elena Boldo, Diana Gomez-Barroso, Gonzalo Lopez-Abente

**Affiliations:** ^1^Department of Environmental Epidemiology and Cancer, National Centre for Epidemiology, Carlos III Institute of Health, Avenida Monforte de Lemos, 5, 28029 Madrid, Spain; ^2^CIBER en Epidemiología y Salud Pública (CIBERESP), 08003 Madrid, Spain; ^3^Division of Medicine, Lancaster University, Lancaster LA1 4YB, UK

## Abstract

Spatial aggregation of different industrial facilities leads to simultaneous release of pollutant emissions. Our objective is to study cancer mortality risk associated with residence in the vicinity of pollutant factories. We used data on industries for year 2007 (3458 facilities). For the 8,098 Spanish towns, we defined a factor with 4 levels based on the number of factories in a radius of 2.5 km from the centroid of each town (industrial factor). We also used data of land cover use to approximate the percentage of municipal land used for industrial activities in each Spanish town (land-used variable). For both variables we fitted Poisson models with random terms to account for spatial variation. We estimated risk trends related with increasing number of factories or percentage of land used for industrial activities. We studied 33 cancer causes. For the industrial factor, 11 causes showed trend associated with increasing factor level. For the land use variable, 8 causes showed statistically significant risks. Almost all tumours related to the digestive system and the respiratory system showed increased risks. Thus mortality by these tumours could be associated to residence in towns nearby industrial areas with positive trend linked to increasing levels of industrial activity.

## 1. Background

Exposure to pollution as a cause of cancer has been frequently studied. Studies using *in vitro *assay systems and biomarkers have proved mutagenic activity in air samples from urban and industrialized areas [1]. Important sources of pollution are emissions from industrial activity. The International Agency for Research on Cancer (IARC) classifies as carcinogens substances and compounds present in air emissions from industrial facilities such as some heavy metals (cadmium, chromium, nickel and arsenic), Volatile Organic Compounds (VOC) benzene or asbestos [2].

Spatial aggregation of factories in industrial estates is a common practice due to legal regulations or economic reasons. This spatial aggregation leads to simultaneous release of pollutant emissions from different industrial facilities over the same area. Potential hazardous effects of those emissions are an important issue from the Public Health point of view and research has been done upto the present date focusing on different diseases and populations, such as cancer, heart disease, mutations in children [1, 3–5]. The availability of direct measures would be the ideal tool for these kinds of studies [6, 7], unluckily in many situations this is still not possible. A more common option is using the geographical location of those facilities to study the population health in their surroundings [8, 9]. 

Risk cancer evidence of living near to pollutant factories and, therefore, being exposed to their pollution is limited. However, some authors have described associations between lung cancer, metallurgical industry, and other industrial areas [10, 11]. Also, lymphomas and leukemia are more frequent in the proximities of industrial areas [12, 13]. In those and other studies, the authors have explored the idea of estimating risk according to distance [14–16]. However, most of these studies based on distances to point sources consider a unique focus or when there is more than one focus, they are individually analysed. Only a few studies approach the multi-source scenario [17, 18]. However, as we mention above, the normal situation is to have more than one pollutant source in the same area.

The implementation of the European Commission directive 96/61/CE (Integrated Pollution Prevention and Control, IPPC) makes compulsory for industries to get the so-called Integrated Environmental Permit to be able to operate. Information gathered as a consequence of the application of these statutory provisions constitutes an inventory of industries with environmental impact in Spain and across Europe. The information is registered by the European Pollutant Release and Transfer Register (E-PRTR); this register makes it compulsory to declare all emissions that exceed the designated thresholds. IPPC and PRTR records thus constitute a public inventory of industries, created by the European Commission, which is a valuable resource for monitoring industrial pollution and, by extension, renders it possible for the association between residential proximity to such pollutant installations and risk of cancer mortality to be studied [11, 19–21].

Another initiative of the European Union supports the European Project Coordination of Information on the Environment CORINE Land Cover 2006 (I&CLC2006) (European Environment Agency). This European database uses satellite images to classify the land cover into 44 classes including land used for industry.

In this paper, we explored potential associations between cancer mortality and number of industries sited in the vicinity of Spanish towns, using the information from the IPPC and PRTR records. A second analysis using land used data was also performed.

## 2. Material and Methods

### 2.1. Industries

**Figure 1:**
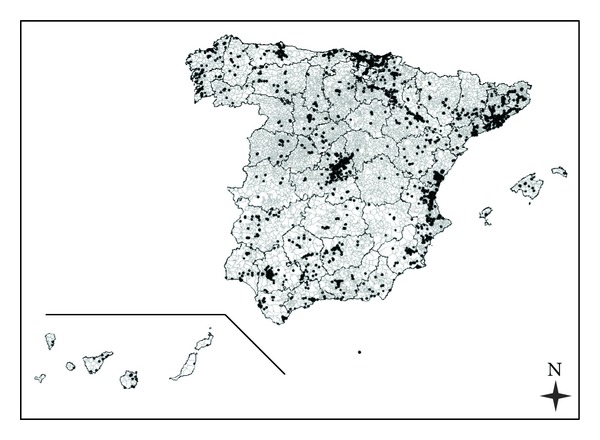
Map with the location of 3458 industrial facilities in black dots.

**Figure 2:**
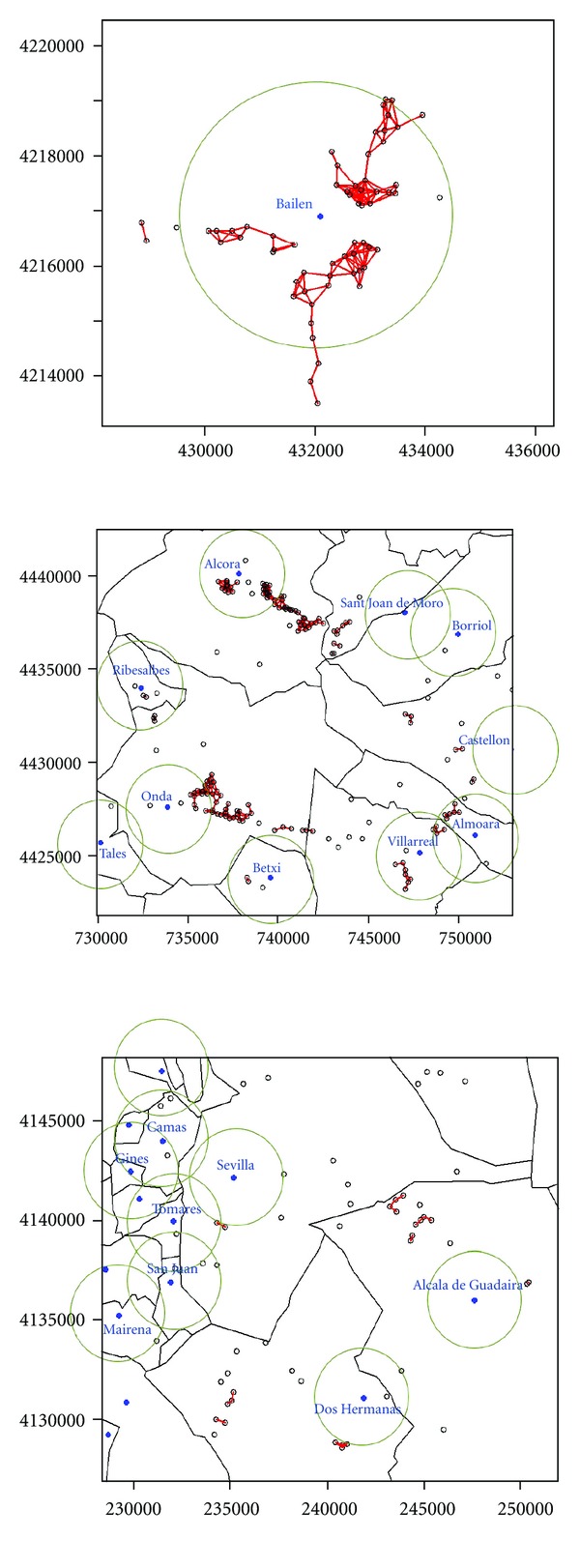
Examples of locations of facilities around towns. Blue dots are the towns centroid. Green rings are the 2.5 km buffer centred in the town centroid. Small black rings are the industrial facilities. Red lines link facilities that are at most 500 m apart from each other.

We used data on industries from the IPPC (Ministry for the Environment and Rural and Marine Habitats—*Ministerio de Medio Ambiente y Medio Rural y Marino*, 2007) to define a variable regarding the magnitude of industries in the vicinity of the towns (*Industry*). Previously, we validated the co-ordinates for all entries, excluding farms, in the inventory of IPPC industries that reported releases to air in 2007. For this analysis, we selected exclusively those facilities with positively validated co-ordinates, 3458 facilities. The following map shows the location of the selected facilities (Figure 1).  Figure 2 shows three examples of locations of some industrial facilities around towns (online version).

For the 8,098 Spanish towns included in the census of 2001, we constructed a new variable “number of industrial facilities in a radius of 2.5 km from the centroid of the town”. We used the centroid of the inhabited area within the town boundaries, not the centroid of the polygon. Using this variable, we built a factor with 4 levels (*Industrial factor)*. We gave the value 0 for those municipalities with no factory within the radius; 1 for those with one factory (small industrial estates); 2 for those with two, three, or four (medium industrial estates); 3 for those with more than four factories (large industrial estates).

From the European Project Coordination of Information on the Environment CORINE Land Cover 2000 (I&CLC2000), we got data of land cover use (European Environment Agency). One of its 44 categories is defined as “industrial or commercial units”. For each Spanish town, we computed the percentage of land in that category (*Land use*). In the analysis, we used 10% as a unit to be able to identify potential effects.

### 2.2. Cancer Association

An ecological study was designed to explore potential association. We analysed 33 causes of cancer mortality at municipal level (8,098 Spanish towns), over the period 1997–2006. Observed municipal mortality data were drawn from the records of the National Statistics Institute (*Instituto Nacional de Estadística*—*INE*) for the study period. Expected cases were calculated by taking the specific rates for Spain as a whole, broken down by age group (18 groups, 0–4, 5–9,…, 85, and over), sex, and five-year period (1997–2001, 2002–2006) and multiplying these by the person-years for each town, broken down by the same strata. To compute the person-years, the two five-year periods were considered, with data corresponding to 1999 and 2004 taken as the estimator of the population at the midpoint of the study period.  Table 1 shows the number of deaths among women, men and for the total number for the mortality causes considered in the analysis.

### 2.3. Models

Poisson regression models with random effects were used to estimate mortality relative risks (RRs). We extended a classical Poisson regression model with random effects to avoid the extra-Poisson variability and to include possible spatial autocorrelation present in the data. We used two different models, a Poisson regression with an unstructured random effect, Mixed model [22], and a Bayesian conditional autoregressive model proposed by Besag, York and Molliè (BYM) [23], that also includes a spatial random effect.

**Mixed Model:**

(1) Oi~Poisson(μi=Eiλi),log⁡(λi)=ρ+αi∗Indi+∑jβjSoci+hi⇒log⁡(μi)=ρ+log⁡(Ei)+αi∗Indi+∑jβjSoci+hi,              hi~Normal(0,τh).

**BYM Model:**

(2) Oi~Poisson(μi=Eiλi),log⁡(λi)=ρ+αi∗Indi+∑jβjSoci+hi+bi,⇒log⁡(μi)=log⁡(Ei)+ρ+αi∗Indi+∑jβjSoci+hi+bi,    hi~Normal(0,τh), bi~Car.Normal(ηi,τb).

In the above models Ind refers to either industry factor or land use variable, Soc are the sociodemographic covariates; *h*_*i*_ is the unstructured random effect; *b*_*i*_ is the spatial random term containing municipal contiguities. For the hyperparameters *τ*_*h*_ and *τ*_*b*_, we assumed LogGamma prior distributions.

Either the *Industry variable* or *land use variable* was included in the models along with the sociodemographic covariates. Relative risks (RRs) and their 95% credible intervals (95% CIs) were estimated for all covariates. Observed deaths (*O*_*i*_) were the dependent variable and expected deaths (*E*_*i*_) were the offset. We then computed trend tests to assess increases in risk with increases in the variable* Industry*. Separate analyses were performed for the overall population (All) and each sex (Women and Men). A final model (Mixed or BYM) for each cancer cause was selected according to Deviance Information Criteria (DIC). According with this criterion, models with smaller DIC should be preferred to models with larger DIC [24].

The socio-demographic indicators (Socs) included were chosen for their availability at municipal level and potential explanatory ability vis-à-vis certain geographic mortality patterns: population size; percentage of illiteracy, farmers and unemployed; average persons per household according to the 1991 census; mean income as a measure of income level [25]. Before their inclusion in the model, indicators were standardised.

The industrial factor was included in the model as a categorical variable (factor) being 0, towns without facilities within the radius, and the reference level. Land use variable was included as continuous variable. We also fitted models with both variables. Integrated nested Laplace approximations (INLAs) were used as a tool for Bayesian inference. For that purpose, we used R-INLA [26] with the option of Gaussian estimation of the parameters, a package available in the R environment [27]. Spatial data on municipal contiguities was obtained by processing the official INE maps.

## 3. Results

### 3.1. Industry

Table 2 shows the frequency of the variable “number of factories within 2.5 km from the centroid of the “town” and the sum of population in relation to the number of facilities. Accordingly to these results, the majority of towns, 85.13%, had no industry within 2.5 km; however in population terms, this percentage was reduced to 56.95%. For the remaining levels, on the contrary, the percentage of towns was lower than the percentage of population. Adding all towns with at least one factory within the radius, we had 1204 towns and a population of 17,302,539.

Level 2 grouped 5.1% of towns and 17.58% of total population, and level 3 1.98% of towns and 9.68% of total population.

Land use variable showed a range of values between 0 and 47.76%, with a mean of 0.5119% and a standard error of 2.59. Number of towns with less than 0.1% of land used for industry was 6916. Spearman's correlation between the industrial factor and the land use variable was 0.57. 

### 3.2. Cancer Association

As we mentioned in Section 2, the selection of the final model between the Mixed model and the BYM model was done using the DIC. Generally causes with low number of cases showed smaller DIC for the Mixed model. (To identify those causes fitted with the Mixed model we added an asterisk, *, by their name.) Table 3 shows the results of the trend test.

**Figure 3:**
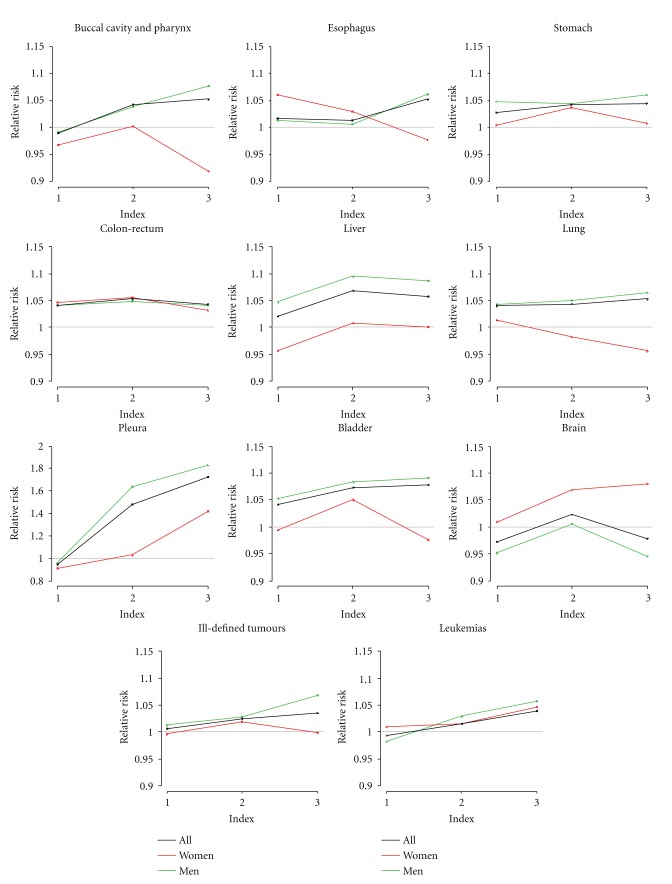
Graphs for the causes that showed trend. Each line resents each of the population groups: black line is for the total population (All), red line is for women, and green for men.

Graphs of the RR according to the levels of the industrial factor for the 10 mortality causes that showed statistically significant trend along with esophagus are in Figure 3. Graphs have 3 lines representing each of the population groups. The black line is for the total population (All), the orange line is for women, and the green for men. Mortality causes in graphs are buccal cavity and pharynx, esophagus, stomach, colon-rectum, liver, lung, pleura, bladder, brain, ill-defined tumours and leukemias.

Results for the analysis with the variable on land used showed that 8 causes were associated with this variable: stomach, liver, lung, pleura, breast, bladder, non-Hodgkin's lymphoma, and leukemias (Table 4). The only cause that did not showed association with the industrial factor was non-Hodgkin's lymphoma. Results for models with the two variables showed that the only risk estimations for the index that changed with the inclusion in the model of the land use variable were risks for L3 (large industrial states). New risk estimations were slightly lower than those in the model with the industrial factor only.

## 4. Discussion

From the 33 studied mortality cancer causes, 11 causes showed positive trend with the industrial factor and/or the land use variable. The most important tumours of the digestive system showed either trend and/or increases in mortality risk: buccal cavity and pharynx, esophagus, stomach, colon-rectum, and liver. Respiratory system cancers that showed trend and risk were lung and pleura. The remaining causes with positive trend tests were bladder, brain, leukemias, non-Hodgkin's lymphoma, and ill-defined tumours. Non-Hodgkin's lymphoma only showed increased risk with the land use variable. Most of the causes, less colon-rectum and brain, showed more risk for men. 

### 4.1. Industry Variable versus Land Used Variable

The use of the geographical information available in the PRTR + IPPC register implied some decisions and assumptions that need to be discussed. For year 2007, these registers had more than 6,000 entries. Our experience with the previous register, EPER, was that recorded co-ordinates were mistaken for numerous facilities [28]; thus, we validated the co-ordinates. The validation process gave us a total of 3458 confirmed locations. We agreed to use only the validated information knowing that we were underestimating the number of pollutant sources; what could bias our results. Then, we decided to construct the variable based on distance between town centroids and factories choosing 2.5 km as maximum. The selection of the maximum distance was another difficult decision due to the variety of industries and pollutants included in this study. The range of distances used in point sources studies vary from 500 m to 35 km depending on the kind of industry and pollutant studied [29], nuclear plants have higher distances, such as 35 km [30], and petrochemical plants have around 3 km; however, when effects were found, it was in short distances [31–33]. The fact of choosing a specific distance directly affects the outcome of the study; therefore, we decided to select one based on positive results of these previous studies knowing that our decision could be another limitation in our findings.

On the other hand, the main limitation using land used data was that, in Spain, as in many countries, land reserved for industrial activities is not exclusively used to allocate industrial facilities and factories, also many warehouses, wholesale warehouse, and facilities for other kinds of activities that do not involve pollutant emissions are allocated on industrial land. The use of this kind of data without a previous examination of the real activities sited in those areas could mislead the results with an overestimation of the number of pollutant sources. Specifically, in our study the number of causes associated to presence of industry was lower when we used the data based on land use, 8 causes, than when we used the data of number of industries, 10 causes; consequently, what could suggest that the overestimation of sources diluted the effect of industry on cancer mortality.

### 4.2. Pollution

Industrial emissions are important contributors for exposure to pollutants, but also occupational exposure, traffic, or indoor exposure contributes substantially. In this paper, we focused only on industrial pollution; thus, our results would only explain part of the total exposure to pollutants. Nevertheless, the study had several strengths too. For example, we were able to include all Spanish towns, covering a total population superior to 40 million, what gives more power to the analysis, as some results showed or the fact that estimated trends and RRs for pleura cancer were the highest, what agrees with the known aetiology for that tumour that says the exposure to asbestos is the main risk factor, being industrial activity the main source of asbestos exposure [34]. In contrast, tumours without evidence or suspected association with environmental pollution such as bones and melanoma did not show association [35].

An alternative method to assess pollution is the use of dispersion models of pollutants [36]. Some authors have used these models to study the health effects of exposure to hazard substances [37]. Nevertheless, the complexity of those models and the demand of information on meteorological and geographical conditions exceeded the purpose of our analysis that was to work with a whole country with an extension of 504030 km^2^ and with 3458 industrial facilities. The same reasons apply to the use of isotropic distance instead of anisotropic. This last decision may introduce bias in the results; however, these problems would, in any event, affect the analysis by restricting the ability to find positive results, shifting the results towards the null hypothesis, rather than providing spurious results.

### 4.3. Cancer

For this study, we used mortality data from the official registers. Unfortunately, at present, there is no nationwide cancer register in Spain. The noninclusion of incidence data is an important limitation on the study of potential risk factors. The lack of information about non-lethal cancer cases may bias the analysis; however, in Spain tumours with lower survival rates are well represented using death certificates according to Pérez-Gómez et al. [38]. Furthermore, we believe there are at most small differences in survival rates or quality of care between regions due to the universal health system established in Spain in 1986.

For this discussion, we aggregated the causes that showed trend in four groups according to their characteristics: (1), digestive system tumours: buccal cavity and pharynx, esophagus, stomach, colon-rectum, and liver; (2), respiratory system and bladder tumours: lung, pleura, and bladder; (3), haematological tumours: leukemias; (4), brain and ill-defined tumours.

#### 4.3.1. Group 1, Digestive System Tumours

The main characteristic for RRs of this group is their positive trend. Associations between these tumours and substances released by industries were reviewed by Clapp et al. in 2008 [39]. This review included papers published between 2005 and 2007 that showed evidence or suspected association between cancer and environmental or occupational exposures; several of these studies considered digestive system tumours [35]. Also, a recent study carried out in Spain showed association between digestive system tumours and residential proximity to metal production and processing facilities [12]. A second important characteristic in our results for mortality risks of digestive system tumours was differences by sex. For towns with small and medium industrial estates (L1 and L2), most of the tumours showed similar estimated mortality risk for women and men, although those for women were in general slightly lower; but, in contrast, RRs for women and men in towns, with large industrial estates were different. For these towns estimated risks for women decreased reaching, in most of the cases, values below 1. This obvious change in the trend for women and men's mortality risks could point out occupational exposures. We consider this hypothesis later in this discussion; however, not much research has been done on this hypothesis so far.

#### 4.3.2. Group 2, Respiratory System and Bladder Tumours

These 3 causes, lung, pleura, and bladder cancer, are also reviewed by Clapp et al. [39]. Again, a recent study carried out in Spain showed association between some respiratory system tumours mortality and residential proximity to combustion installations [19]. By tumours, lung cancer, with estimated increases in mortality risks for total population and men equal or superior to 4%, is the most studied cancer in relation with exposure to pollutants and many studies confirm associations with metals, pesticides, solvents, or air pollution [39]. Regarding bladder cancer, in 2006, a paper studying mortality and mining industry in Spain showed positive association [40]. Internationally, many occupational studies provide results associating this tumour with industrial activity [41]. For pleura cancer, the strong causal role of asbestos has been shown in many studies [34, 42], what supports the association with an increasing number of industries in the vicinity of towns showed in our study. This specific result showed the reliability of our analysis.

#### 4.3.3. Group 3, Haematological Tumours

Among the haematological tumours, leukemias showed trend for men. Associations between haematological tumours and industry have been the motivation for several studies. In some countries such as the UK, Spain, and Germany, they have been associated with nuclear plants and incinerators [16, 43, 44]. Again, a recent study carried out in Spain showed association between leukemias mortality and residential proximity to metal production and processing installations [45]. Many occupational studies associated leukemias with industrial exposure [39, 46].

#### 4.3.4. Group 4, Brain and Ill-Defined Tumours

These two causes showed statistically significant trend with the industrial factor. Brain cancer showed stronger association with women while ill-defined tumours association among men. The positive association for brain cancer mortality among women is a quite surprising result and should be studied further because the high risk areas for female mortality match moderately with industrialized areas. The review of the literature did not show an explanation for this increased risk [35, 47]. Ill-defined tumours showed similar risks to digestive system cancers.

#### 4.3.5. Sex Differences

All the associated causes, less colon-rectum, showed different behaviour for men and women. Causes showing more association for men's mortality were buccal cavity and pharynx, stomach, lung, pleura, bladder, ill-defined tumours and leukemias, while only brain cancer morality showed higher risk for women. These differences could be related with two main factors that have distinctive impact for men and women. One is tobacco smoke and the other is occupation. In Spain the number of female smokers has traditionally been lower than the number of male smokers [48]. This differential smoking pattern is very obvious looking at the number of deaths by tobacco-related causes in men and women [49]. The second possible explanation is occupation. In Spain, according to the official historical series of employed population from 1976 until 2008, for most of the period, only one in five industry workers was a woman (Office for National Statistics, INE) [50].

Due to the characteristics of this study, especially the data, we were not able to study exposure to specific pollutant substances and compounds, what would have yielded a more conclusive approach. Nevertheless, we would like to point out that the higher the number of industries close to residential areas, the higher the probability of more kind of pollutants population is exposed to, what contribute the multifactorial process of cancer causation [51].

## 5. Conclusion

Cancer mortality for digestive and respiratory system tumours showed increases in risk associated with increasing number of industrial facilities in the vicinity towns. Further study is needed to confirm these initial results. Countries or regions with similar registers of pollutant industries could perform similar analyses; those from the European Union could use directly the PRTR register.

## Figures and Tables

**Table 1:** Number of deaths for 33 cancer causes, ICD 9 and ICD 10 code, women, men and total, Spain, 1997–2006.

Tumours	ICD 9	ICD 10	Women	Men	Total
Buccal cavity and pharynx	140–149	C00–14	3,842	18,136	21,978
Esophagus	150	C15	2,383	15,377	17,760
Stomach	151	C16	22,917	36,754	59,671
Small intestine	152	C17	620	744	1,364
Colon-rectum	153-154	C18–20	52,746	68,095	120,841
Liver	155	C22	6,646	17,609	24,255
Gall-bladder	156	C23	8,785	4,682	13,467
Pancreas	157	C25	19,590	22,328	41,918
Peritoneum	158	C45.1, C48	1,281	1,066	2,347
Nasal fossae	160	C30, C31	272	636	908
Larynx	161	C32	623	16,674	17,297
Lung	162	C34	20,923	160,104	181,027
Pleura	163	C38.4, C45.0	618	1,538	2,156
Bones	170	C40	1,219	1,702	2,921
Connective tissue	171	C41	2,034	2,148	4,182
Melanoma	172	C43	3,414	3,987	7,401
Skin	173	C44, C46	2,134	2,498	4,632
Breast	174	C50	57,830		57,830
Other uterus	179–181	C53-54	3,355		3,355
Uterus	182	C55	18,080		18,080
Ovary	183	C56	18,046		18,046
Prostate	185	C61		55,772	55,772
Testis	186	C62		425	425
Bladder	188	C67	7,175	34,107	41,282
Kidney	189	C64	5,809	11,532	17,341
Brain	191	C71	10,067	12,622	22,689
Other tumours nervous sys	191	C72	401	400	801
Thyroid	193	C73	1,800	911	2,711
Ill-defined tumours	195–199	C76–C80, C96	26,670	33,968	60,638
Non-Hodgkin's lymphoma	200, 202	C82–84	11,109	12,229	23,338
Hodgkin's	201	C81	1,034	1,345	2,379
Myeloma	203	C90	7,637	7,541	15,178
Leukemias	204–208	C91–95	12,775	16,295	29,070

Total	140–208	C00–C97	331,835	561,225	893,060

**Table 2:** Industry levels, number of facilities, number of towns within the radius, population.

Level	No. facilities	Towns	Population
L0	0	6894 (85.13%)	22890314 (56.95%)
L1	1	630 (7.78%)	6344330 (15.78%)
L2	2	228 (2.82%)	3318915 (8.26%)
L2	3	121 (1.49%)	2684455 (6.68%)
L2	4	64 (0.79%)	1062607 (2.64%)
L3	5	41 (0.51%)	847854 (2.11%)
L3	6	32 (0.40%)	771036 (1.92%)
L3	7	17 (0.21%)	209541 (0.52%)
L3	8	21 (0.26%)	324714 (0.81%)
L3	9	6 (0.07%)	62709 (0.16%)
L3	10	8 (0.10%)	347082 (0.86%)
L3	11	9 (0.11%)	181530 (0.45%)
L3	12	7 (0.09%)	449401 (1.12%)
L3	13	2 (0.02%)	35078 (0.09%)
L3	14	4 (0.04%)	163150 (0.41%)
L3	15	4 (0.04%)	331421 (0.82%)
L3	16	3 (0.04%)	101026 (0.25%)
L3	18	3 (0.01%)	15151 (0.04%)
L3	25	1 (0.01%)	18854 (0.05%)
L3	27	1 (0.01%)	8934 (0.02%)
L3	28	1 (0.01%)	7269 (0.02%)
L3	55	1 (0.01%)	17482 (0.04%)

**	Total	8098 (100%)	40192853 (100%)

**Table 3:** Trend test results. Trend and credible intervals.

Tumours	All		Women		Men	
	Trend	(2.5%, 97.5%)	Trend	(2.5%, 97.5%)	Trend	(2.5%, 97.5%)
Buccal cavity and pharynx	**1.03**	**(1.00, 1.05)**	0.98	(0.95, 1.02)	**1.03**	**(1.01**, **1.06)**
Esophagus	**1.03**	**(1.01, 1.06)**	0.99	(0.93, 1.04)	**1.04**	**(1.01**, **1.07)**
Stomach	**1.02**	**(1.01, 1.04)**	1	(0.98, 1.02)	**1.04**	**(1.02**, **1.06)**
Small intestine*	0.96	(0.89, 1.03)	0.99	(0.90, 1.08)	0.97	(0.89, 1.05)
Colon-Rectum	**1.03**	**(1.02, 1.04)**	**1.01**	**(1.00, 1.03)**	**1.03**	**(1.02**, **1.05)**
Liver	**1.06**	**(1.04, 1.09)**	1.03	(0.99, 1.08)	**1.07**	**(1.04**, **1.10)**
Gall-Bladder	**1.02**	**(1.00, 1.05)**	1.01	(0.98, 1.04)	**1.03**	**(1.00**, **1.07)**
Pancreas	1	(0.99, 1.02)	0.99	(0.98, 1.01)	1	(0.99, 1.02)
Peritoneum*	1.03	(0.98, 1.08)	1.03	(0.97, 1.10)	1.03	(0.96, 1.11)
Nasal fossae*	0.99	(0.93, 1.07)	0.96	(0.84, 1.09)	1.01	(0.92, 1.10)
Larynx	1.01	(0.99, 1.04)	0.99	(0.91, 1.09)	1.01	(0.99, 1.04)
Lung	**1.04**	**(1.03, 1.05)**	0.97	(0.95, 0.99)	**1.04**	**(1.03**, **1.06)**
Pleura*	**1.21**	**(1.14, 1.28)**	**1.09**	**(1.00, 1.19)**	**1.25**	**(1.16**, **1.33)**
Bones	0.97	(0.93, 1.01)	1.02	(0.96, 1.09)	0.94	(0.89, 0.99)
Connective tissue*	1.01	(0.97, 1.05)	0.99	(0.94, 1.05)	1.02	(0.98, 1.07)
Melanoma	0.96	(0.94, 0.99)	0.96	(0.92, 1.00)	0.97	(0.93, 1.00)
Skin	0.93	(0.89, 0.97)	0.93	(0.88, 0.99)	0.94	(0.89, 0.99)
Breast			1	(0.99, 1.01)		
Other uterus*			1.02	(0.98, 1.06)		
Uterus			0.98	(0.95, 1.00)		
Ovary			1.01	(0.99, 1.03)		
Prostate					0.99	(0.98, 1.01)
Testis*					1	(0.98, 1.01)
Bladder	**1.04**	**(1.02, 1.06)**	1.01	(0.98, 1.04)	**1.05**	**(1.03, 1.07)**
Kidney	**1.03**	**(1.01, 1.05)**	**1.04**	**(1.01, 1.07)**	**1.02**	**(1.00, 1.05)**
Brain	**1.01**	**(0.99, 1.02)**	**1.03**	**(1.00, 1.05)**	0.99	(0.97, 1.01)
Other tumours nervous sys*	0.99	(0.91, 1.07)	1	(0.89, 1.12)	0.98	(0.88, 1.09)
Thyroid	1.01	(0.96, 1.06)	1.02	(0.97, 1.08)	0.99	(0.92, 1.06)
Ill-defined tumours	**1.02**	**(1.01, 1.04)**	1	(0.98, 1.01)	**1.04**	**(1.02, 1.06)**
Non-Hodgkin's lymphomas	1.01	(0.99, 1.03)	1	(0.98, 1.03)	1.01	(0.98, 1.03)
Hodgkings*	0.98	(0.94, 1.03)	0.99	(0.93, 1.06)	0.97	(0.92, 1.03)
Myeloma*	1.01	(0.99, 1.03)	1.02	(0.99, 1.04)	1	(0.98, 1.03)
Leukemias	**1.02**	**(1.01, 1.04)**	1.02	(0.99, 1.04)	**1.02**	**(1.00, 1.04)**

*Indicates if the fitted model was the Mixed model.

**Table 4:** Relative risks and credible intervals of the variable of land use.

Tumours	All		Women		Men	
	RR	(2.5%, 97.5%)	RR	(2.5%, 97.5%)	RR	(2.5%, 97.5%)
Stomach	**1.02**	**(1.00**, **1.04)**	1.01	(0.98, 1.04)	**1.04**	**(1.01**, **1.07)**
Liver	**1.05**	**(1.00**, **1.10)**	1.06	(0.99, 1.14)	**1.05**	**(1.00**, **1.11)**
Lung	**1.03**	**(1.00**, **1.05)**	0.98	(0.94, 1.02)	**1.03**	**(1.01**, **1.06)**
Pleura*	**1.18**	**(1.09**, **1.31)**	**1.16**	**(1.06**, **1.39)**	**1.2**	**(1.09**, **1.33)**
Breast			**1.04**	**(1.00**, **1.07)**		
Bladder	**1.04**	**(1.00**, **1.07)**	1.01	(0.96, 1.06)	**1.04**	**(1.00**,**1.07)**
Ill-defined tumours	1.02	(0.99, 1.04)	0.99	(0.96, 1.02)	**1.04**	**(1.01**,**1.07)**
Non-Hodgking's lymphomas	**1.05**	**(1.01**, **1.08)**	1.03	(0.99, 1.08)	**1.06**	**(1.01**, **1.10)**
Leukemias	**1.03**	**(1.00**, **1.06)**	**1.02**	**(1.00**, **1.06)**	**1.04**	**(1.01**, **1.08)**

*Indicates if the fitted model was the Mixed mode.
